# Molecular Excitons and Plasmons in Acenes and Their Radical Cations

**DOI:** 10.1002/jcc.70211

**Published:** 2025-08-18

**Authors:** Anna M. Weidlich, Andreas Dreuw

**Affiliations:** ^1^ Interdisciplinary Center for Scientific Computing Ruprecht‐Karls University Heidelberg Germany

**Keywords:** acenes, excited states, excitons, open‐shell systems, plasmons

## Abstract

For existing and potential applications of acenes and acene derivatives, properties of their excited states play a central role. In describing these, the molecular orbital picture can reach its limits, and consideration within the quasi‐particle picture can enable further insight. In this work, exciton size ($d_{exc}$), hole and electron size ($\sigma_{h}$ and $\sigma_{e}$) and correlation coefficient ($R_{eh}$) of excited states of acenes and acene cations are investigated using TD‐DFT at the TDA/CAM‐B3LYP/6‐311G* level, with a focus on their development with increasing acene length. Furthermore, employing a previously established approach, it is shown that the electronic structure of the ^1^B_b_ and ^2^B_b_ states of neutral and cationic anthracene can be understood as molecular plasmons.

## Graphical abstract

The electronic structure and the physical character of the energetically low‐lying excited electronic states determine the applicability of acenes and their radical cations in organic electronics. Here we employ the intuitive picture of molecular excitons and plasmons to classify these states and their properties with increasing lengths of the acenes to obtain valuable insights into their electronic structure.

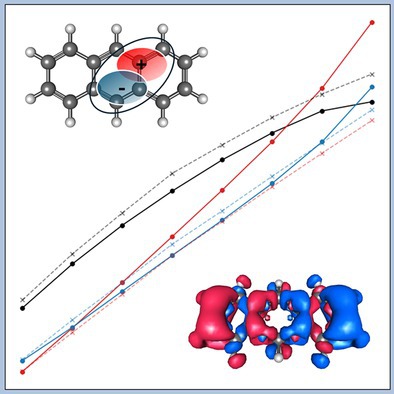

## 1 Introduction

Acenes are an extensively studied molecular class for both their electronic structure in general, as well as their existing and potential applications [1, 2, 3, 4, 5]. At present, the most interesting application is their use as organic materials in electronic devices, where the properties of low‐lying electronic excited states determine the suitability for different applications [6, 7, 8, 9, 10]. In the field of organic materials, two different concepts of describing excited states are encountered, that is, the quantum‐chemical description of electronic excited states of a single molecule and the description of an excited state in an extended solid from solid‐state physics. The latter uses the concept of quasi‐particles to treat the collective behavior of particles, as if they were one single particle. Thereby, the many‐body problem of strongly interacting electrons and nuclei within the solid is reduced to the problem of one particle that is weakly interacting with the solid. For example, an electron or an electron‐hole that moves through the solid can be described by the respective electron and hole quasi‐particles. Electronic excited states in this picture are described as excitons, which are an electron and a hole quasi‐particle bound together. A special case of an exciton, which is a collective excitation, where all the electrons oscillate simultaneously, is called a plasmon. Commonly employed methods here are the GW approximation (GW) in combination with the Bethe‐Salpeter equation (BSE), which have been successfully employed to describe electronic excitations in solid‐state and gas‐phase organic molecules, including acenes [11, 12, 13, 14, 15, 16, 17, 18, 19, 20].

While excitation energies and the character of electronic transitions are easily accessible using quantum‐chemical methods, exciton properties are more elusive in this framework. The consideration of excited states within the quasi‐particle picture can therefore provide valuable information, such as the exciton size, the correlation of electron and hole, and the characterization as either a single‐particle or a plasmonic excitation. Beyond that, the exciton picture also provides an illustrative framework for describing excited‐state properties, where the molecular orbital picture reaches its limits—for example, when an excited state is described by more than one orbital transition. However, the description of excitons and plasmons within wavefunction‐ and density‐based methods for single molecules is not straightforward, and the methodology to achieve this will be discussed in the following [21, 22, 23, 24, 25].

The absorption spectra of acenes are well‐characterized from an experimental as well as a quantum‐chemical point of view. The characteristic p‐, α‐ and β‐bands can be shown, using excited‐state methods, to originate from the ^1^L_w_, ^1^L_s_ and ^1^B_b_ excited states, respectively [1, 2, 26]. Here, a revised nomenclature for the excited states ^1^L_a_ (now ^1^L_w_) and ^1^L_b_ (now ^1^L_s_) is used, where the subscript refers to weak and strong electron‐hole correlation as a reliable physical criterion for their unambiguous classification in acene derivatives [26]. In previous work, several bright low‐lying excited states of radical cationic acenes were analyzed within the molecular orbital picture and were set into relation to their neutral counterparts [27]. Furthermore, the development of their excitation energies with growing acene size was investigated. Here, their characterization is extended to include the development of exciton size, electron and hole size, and electron and hole correlation with increasing acene size. Also, the plasmonic character of the ^1^B_b_ and ^2^B_b_ states of neutral anthracene and its cation, respectively, is investigated.

## 2 Theoretical Framework and Recapitulation

### 2.1 Excitons in Molecules

For molecular systems, the exciton wavefunction can be identified with the one‐particle transition density matrix (1TDM) between the ground ($\Psi_{0}$) and excited state ($\Psi_{I}$) [21]. Here, the ground state describes the hole component, and the excited state describes the electron component. This results in a two‐particle wavefunction describing the exciton

(1) $$\begin{array}{c} \begin{array}{ll} & \chi_{exc}(r_{h},r_{e})\\ & =\int \text{⋯}\int \Psi_{0}(r_{h},r_{2},\text{…},r_{N})\Psi_{I}(r_{e},r_{2},\text{…},r_{N})dr_{2}\text{⋯}dr_{N}\; \end{array} \end{array}$$

where N is the number of electrons and $r_{i}$ are the spatial‐spin coordinates. This wavefunction can now be used to extract the exciton properties of excited states [21, 22]. The exciton size describes the distance and spatial extension of the electron and the hole using their respective centroids ${\vec{x}}_{e}$ and ${\vec{x}}_{h}$. Their positions are given as the expectation values of the position operator $\hat{x}$. The exciton size is then defined as the root‐mean‐square (rms) separation between the instantaneous hole and electron positions

(2) $$d_{exc}=\sqrt{{\left\langle |\vec{x_{h}}-\vec{x_{e}}{|}^{2}\right\rangle }_{exc}}$$

For the size of the electron and hole, the variance of their spatial extension around the centroids is computed as

(3) $$\sigma_{h}=\sqrt{{\left\langle {\vec{x_{h}}}^{2}\right\rangle }_{exc}-{\left\langle \vec{x_{h}}\right\rangle }_{exc}^{2}}$$

and

(4) $$\sigma_{e}=\sqrt{{\left\langle {\vec{x_{e}}}^{2}\right\rangle }_{exc}-{\left\langle \vec{x_{e}}\right\rangle }_{exc}^{2}}$$

Additionally, the correlation between the electron and hole can be evaluated, which describes how their spatial distributions depend on each other. Here, a positive value means that the electron and hole are correlated and move together, while a negative value means that they are anti‐correlated and avoid each other. The normalized electron‐hole correlation coefficient is defined as

(5) $$R_{eh}=\frac{{\left\langle \vec{x_{h}}\cdot \vec{x_{e}}\right\rangle }_{exc}-{\left\langle \vec{x_{h}}\right\rangle }_{exc}\cdot {\left\langle \vec{x_{e}}\right\rangle }_{exc}}{\sigma_{h}\sigma_{e}}$$

so that $R_{eh}$ is the normed covariance with respect to the electron and hole sizes. Using these quantities, electronic excited states can be analyzed regarding their exciton properties based on the 1TDM.

### 2.2 Plasmons in Molecules

In extended systems, plasmons arise as collective oscillations of the electron density and can be described using classical electrodynamics. Within the quantum‐mechanical picture, however, the definition of a plasmon is not straightforward, and many approaches to identifying molecular plasmons have been explored. As a result, it has been shown using various procedures that electronic excitations of metal nanoparticles and molecular systems can have plasmonic character [24, 25, 28, 29, 30, 31, 32, 33, 34, 35, 36, 37, 38].

One approach is to define the properties of molecular plasmons using the random‐phase approximation (RPA) for the homogeneous electron gas [24]. The most important criterion here is that plasmons are a linear combination of the *m* possible particle‐hole transitions with the same momentum transfer **q**. They have the highest excitation energy of all the excitations made up from the same *m* transitions and the largest transition strength.

Within the model of the homogeneous electron gas in a box ($V=L^{3}$), the single‐particle states $\psi_{k\sigma }$ are given as

(6) $$\psi_{k,\gamma }(x,s)=\phi_{k}(x)\gamma (s)=\frac{1}{\sqrt{V}}e^{ikx}\gamma (s),\;\gamma \in \{\alpha ,\beta \}$$

with a spatial orbital $\phi_{k}$ and a spin‐function γ(s). The Cartesian and spin coordinates are denoted by x and s, respectively. The wave vector **k** (momentum vector) is given, using periodic boundary conditions, as

(7) $$k_{i}=\frac{2\pi n_{i}}{L}\;(i=x,y,z;n_{i}=0,\pm 1,\pm 2,\text{…})$$

The momentum transfer of a particle‐hole transition is defined as **q**=**j**‐**k**, where **k** and **j** are the wavevectors of the particle‐ and hole‐states, respectively.

Plasmonic excitations were shown to arise in linear polyenes by viewing the π‐electrons as a quasi one‐dimensional electron gas and assigning wavevectors to the Hartree‐Fock (HF) π‐orbitals [24]. This provides a useful qualitative measure to classify the momentum transfer of the particle‐hole excitations. Since in the one‐dimensional model, the wave vectors have only one component, the momentum transfer can be directly related to the corresponding change in the quantum number Δn of the orbitals involved, which is equal to the change in the number of nodes of the involved orbitals. It was further pointed out that this procedure should be applicable to non‐linear systems, if a suitable electron gas model is used [24].

### 2.3 Excited States of Acenes and Acene Cations

Absorption spectra of neutral acenes display three characteristic peaks, the p‐, α‐, and β‐bands [2]. The p‐band is the first excitation for all acenes, except naphthalene, and has a medium absorption intensity. Of the three peaks, the β‐band has the highest excitation energy and the strongest absorption intensity, while the α‐band is almost dark. The corresponding excited states are ^1^L_w_, ^1^L_s_ and ^1^B_b_ for the p‐, α‐ and β‐bands, respectively. The excited state ^1^L_w_ is of B_2u_ symmetry and the excited states ^1^L_s_ and ^1^B_b_ are of B_3u_symmetry.

**FIGURE 1:**
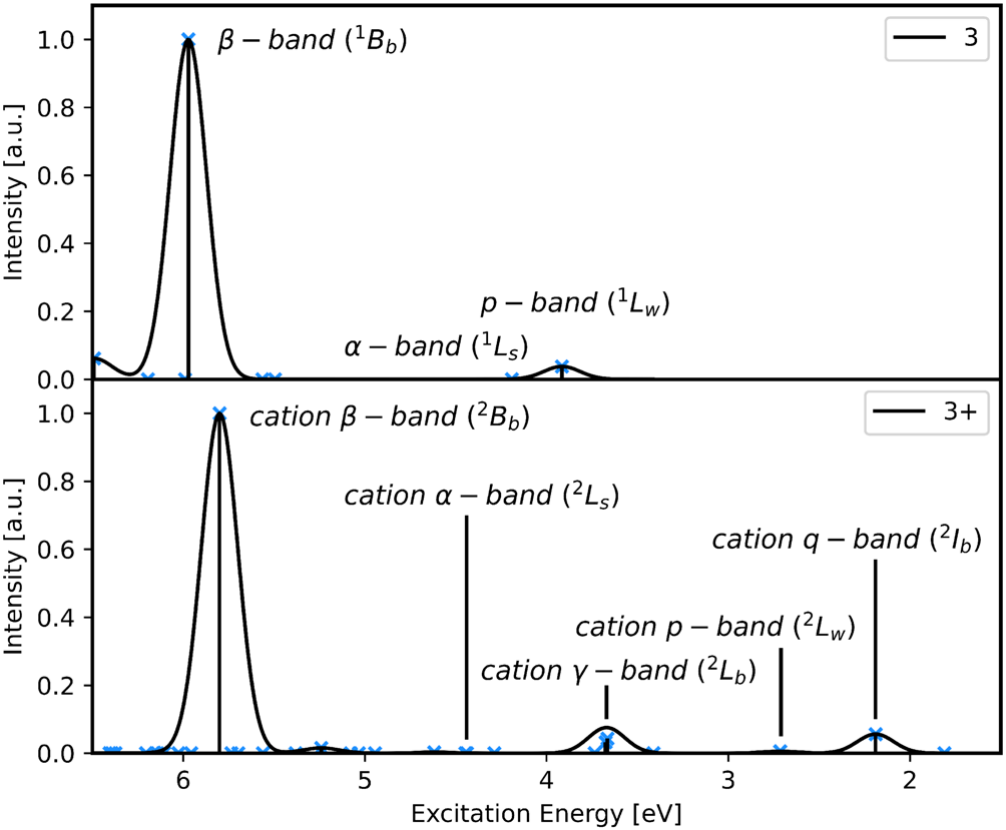
Simulated absorption spectra of **3** (top) and **3^+^** (bottom) obtained by convolution of excitation energies (TDA/CAM‐B3LYP/6‐311G*) using a Gaussian broadening function with a standard deviation of 0.1 eV.

In previous work, excited states of acene radical cations were characterized and set into relation to those of neutral acenes [27]. Excited states of acene cations that are of the same character as the ^1^L_w_, ^1^L_s_ and ^1^B_b_ states were labeled ^2^L_w_, ^2^L_s_ and ^2^B_b_, respectively. Their corresponding absorption bands were named cation p‐, cation α‐, and cation β‐bands, respectively. The relation of neutral to cation excited states was analyzed based on the molecular orbitals participating in the transitions; however, they can also be identified based on their symmetry, excitation energy, oscillator strength, and transition density. The excited states ^2^L_w_, ^2^L_s_, and ^2^B_b_ show the same energetic order and relative absorption intensities as their neutral counterparts. In acene cations, the symmetry of the ground state is A_u_ for an even number of rings and B_3g_ for an odd number of rings. Excited states of the same character, therefore, belong to different irreducible representations depending on whether the number of rings is even or odd. The excited state ^2^L_w_ is of B_2g_/B_3g_ symmetry and the excited states ^2^L_s_ and ^2^B_b_ are of B_3g_/A_u_ for even/uneven number of rings. Further, the excited states ^2^I_b_ and ^2^L_b_ were identified as low‐lying excited states that show a medium absorption intensity, which are not present in the neutral acenes. The excited state ^2^I_b_ is the first excited state for acene cations up to tetracene. The ^2^L_b_ state has higher excitation energies than both ^2^I_b_ and ^2^L_w_, but lower excitation energies than ^2^L_s_ and ^2^B_b_. The corresponding absorption peaks were labeled cation q‐ and cation γ‐bands, and irreducible representations are B_3g_/A_u_ for even/uneven number of rings, respectively. All these excited states and the corresponding absorption bands can be seen in Figure 1 for anthracene and the radical anthracene cation, where they are labeled **3** and **3^+^**, respectively, according to the number of conjugated rings.

**FIGURE 2:**
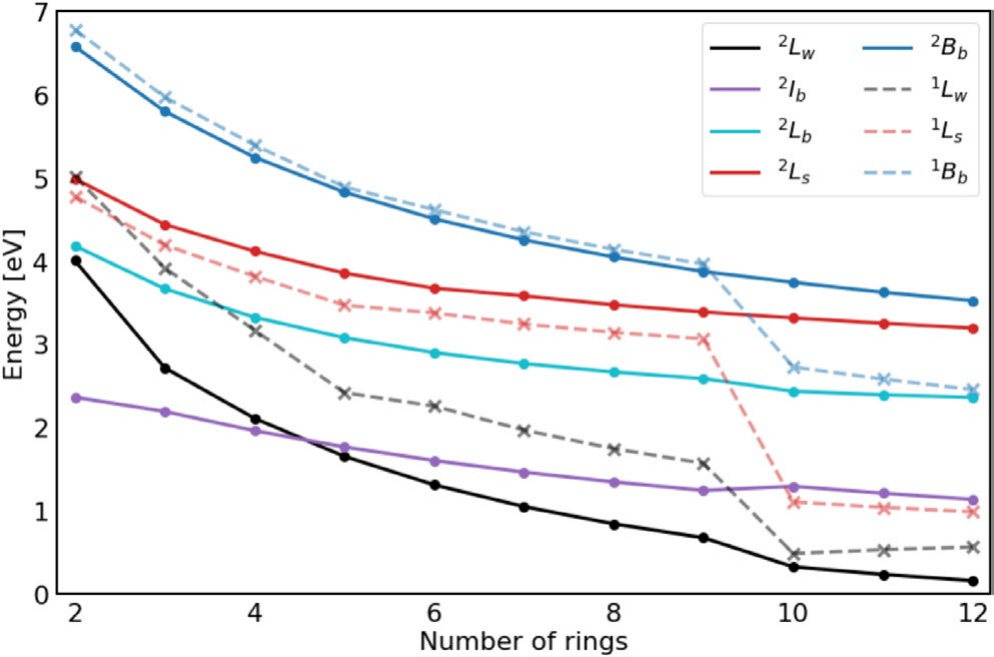
Excitation energies of selected excited states of **2^+^**–**12^+^** (solid lines) and **2**–**12** (dotted lines) at TDA/CAM‐B3LYP/6‐311G*.

It was further investigated how the excitation energy of these excited states changes with growing acene length, which is shown for the mentioned states in Figure 2. A table of excitation energies can be found in the Supporting Information in Table S1. It can be seen and was previously discussed that the theoretical description for both neutral and cationic acenes suffers from increasing length. This is observed in the discontinuities in excitation energies when going from **9**/**9^+^** to **10**/**10^+^** and can be explained by the inability of (TD‐)DFT to properly describe systems with increased multi‐reference ground state character.

## 3 Methodology

All calculations were performed using the Q‐Chem 5.2 program package [39]. Acenes of increasing length from naphthalene to dodecacene and their respective radical cations are investigated and will be named according to the number of conjugated rings, for example, naphthalene is **2** and its cation is **2^+^**. The geometries of all neutral and cationic molecules were obtained at the CAM‐B3LYP(D3(BJ))/6‐311G* level of theory [40, 41, 42]. The $\left\langle \hat{S^{2}}\right\rangle$‐values of **2^+^** to **12^+^** are 0.78, 0.78, 0.79, 0.79, 0.80, 0.80, 0.80, 0.81, 0.82, 0.83, and 0.84, respectively. The stationary points were confirmed to be minima by harmonic frequency analyses, and it was ensured that all geometries were of D_2h_ symmetry. The molecules were oriented so that the x‐axis lies along the long molecular axis, the y‐axis along the short molecular axis, and the z‐axis lies out of plane. This orientation is common in literature and adopted by Q‐Chem; however, it does not conform to the Mulliken convention. The low‐energy singlet/doublet electronic excited states were calculated using the CAM‐B3LYP functional, the 6‐311G* basis set, and employing the Tamm‐Dancoff approximation (TDA). This choice is based on a benchmark in previous work [27]. Also, for a physically correct description of the exciton properties, a long‐range separated xc‐functional is mandatory, since otherwise the particle‐hole attraction is not correctly described [43, 44]. For acenes larger than **9**, the lowest triplet becomes lower in energy than the lowest singlet, leading to triplet instabilities. Meanwhile, for acene cations larger than **9^+^**, the SCF converges to an unstable solution, which is remedied by switching the α‐HOMO and α‐LUMO in the initial guess. Both these issues are discussed in more detail in previous work [27]. Exciton properties [21, 22] (exciton, hole, and electron size and electron‐hole correlation), Kohn‐Sham orbitals, and transition densities were computed as implemented in Q‐Chem and visualized using IQmol Version 2.8.0.

## 4 Results and Discussion

### 4.1 Exciton Properties of Acene and Acene Cation Excited States

The exciton properties of the excited states ^1^L_w_, ^1^L_s_ and ^1^B_b_ for neutral acenes and ^2^I_b_, ^2^L_w_, ^2^L_b_, ^2^L_s_ and ^2^B_b_ for cationic acenes are investigated with respect to exciton size ($d_{exc}$), hole and electron size ($\sigma_{h}$ and $\sigma_{e}$) and electron‐hole correlation ($R_{eh}$). Here, TDA the energetically lowest‐lying excited states ^1^L_w_, ^2^L_w_ and ^1^I_b_ will be discussed in detail. The results for the other excited states can be found in the Supporting Information (Figures S1–S3). The previously mentioned discontinuity in excitation energies going from **9** to **10** and **9^+^** and **10^+^** can be observed as well in the exciton properties for all the investigated excited states, further showing that the states are not well described using linear‐response TD‐DFT‐based methods. For this reason, the results for the molecules **10**–**12** and **10^+^**–**12^+^** are not included here. The plots, including all states, can be found in the Supporting Information (Figures S4–S8).

**FIGURE 3:**
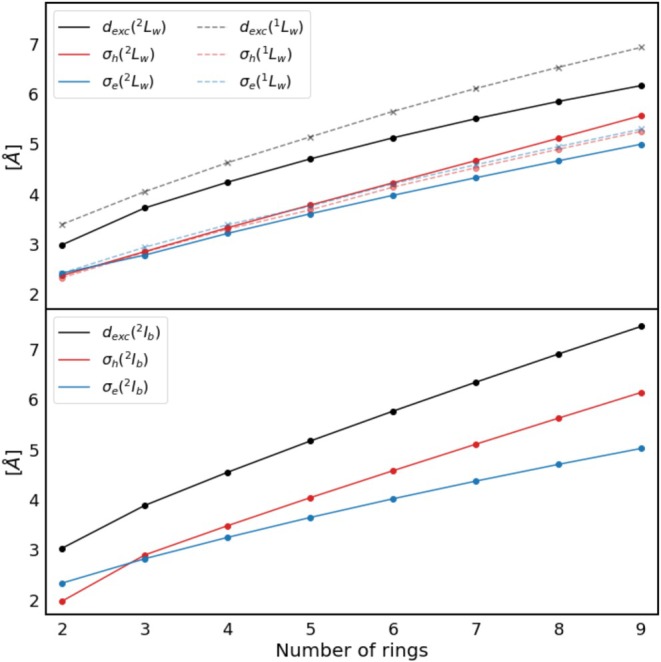
Exciton ($d_{exc}$), hole ($\sigma_{h}$), and electron size ($\sigma_{e}$) of the excited states ^2^L_w_, ^1^L_w_ (top), and ^2^I_b_ (bottom) of **2^+^**–**9^+^** and **2**–**9** calculated using CAM‐B3LYP/6‐311G*.

Figure 3 shows the exciton, hole and electron size of the ^1^L_w_ state of neutral acenes and the ^2^L_w_ state of cationic acenes for **2**–**9** and **2^+^**–**9^+^**. Looking at the ^1^L_w_ state first, the exciton size increases from 3.4 Å to 7.0 Å for **2**–**9**. The hole and electron size show a similar trend starting from 2.3/2.4 Å and going to 5.2/5.3 Å for **9**. For the excited state ^2^L_w_, the exciton size increases from 3.0 Å for **2^+^** to 6.2 Å for **9^+^**. The respective hole and electron sizes are similar for the smaller acene cations and then diverge as the length increases, since the hole size increases faster than the electron size. The hole size is 2.4 Å for **2^+^** and increases to 5.6 Å for **9^+^**, while the respective electron size is 2.4 Å and 5.0 Å.

The exciton properties of the excited state ^2^I_b_ of **2^+^**–**9^+^** are also shown in Figure 3. The exciton size increases from 3.0 Å for **2^+^** to 7.5 Å for **9^+^**. Hole and electron size increase as well, starting from 2.0 Å and 2.3 Å for **2^+^**. The hole size again increases faster so that it is larger than the electron size for **3^+^**. The values for **9^+^** are 6.1 Å and 5.0 Å, respectively for hole and electron size.

**FIGURE 4:**
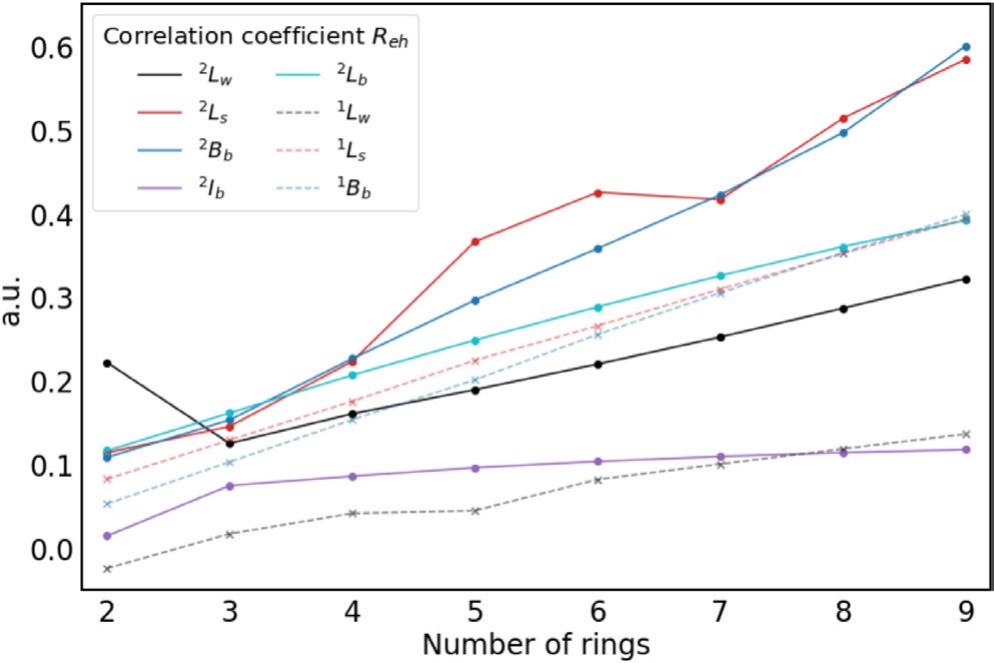
Correlation coefficient ($R_{eh}$) of all investigated excited states of **2**–**9** and **2^+^**–**9^+^** calculated using CAM‐B3LYP/6‐311G*.

Several trends can be observed when looking at the development of the exciton properties of the investigated excited states with increasing acene length. An overall increase in the exciton, hole, and electron size is observed for all investigated states. The exciton size of the excited states of neutral acenes is generally larger than the one of the cations, except for ^2^L_s_ of **6^+^** and **7^+^**, which might however be due to mixing of these states with another excited state (see Supporting Information). An explanation for the smaller exciton size of the cations could be that the electron density contracts when an electron is removed. It would, however, be expected that this effect decreases with the growing number of electrons; however, the exciton size of the cations stays consistently smaller. Also, the hole and electron sizes are not smaller for the cations than for the corresponding neutral acenes. The reason for the smaller exciton size is the stronger correlation between hole and electron, which can be seen in Figure 4.

For the neutral acenes, the electron size is always slightly larger than the hole size, which is also the case for all the excited states of **2^+^** (and ^2^L_s_ of **3^+^** and **4^+^**). Since the hole size increases faster than the electron size in the excited states of the cations, the hole size is generally larger than the electron size for all other acene cations. An explanation for the stronger increase in the hole size as opposed to the electron size could be the stronger delocalization of the already existing positive charge with the increasing size of the π‐system.

One can distinguish between two different developments, where for one set of states, ^1^L_w_, ^2^L_w_ and ^2^I_b_, the exciton, hole and electron size all seem to converge towards a certain value and for the other set of states, ^1^L_s_, ^2^L_s_, ^1^B_b_, ^2^B_b_ and ^2^L_b_, the hole and electron size increase more strongly than the exciton size. It is apparent that, within the molecular orbital picture, the former states are all mainly described by a single transition, while the latter are mainly described by at least two transitions.

This trend can be directly linked to the development of the correlation coefficient ($R_{eh}$), which is shown for all investigated states of **2**–**9** and **2^+^**–**9^+^** in Figure 4. The correlation coefficient increases for all states with increasing acene size. Some irregularities can be observed for the ^2^L_w_ state of **2^+^** and the ^2^L_s_ states of **5^+^**– **7^+^**. A possible explanation is the mixing of these states with other energetically close states. This was observed previously for the ^2^L_w_ state of **2^+^** and is also the case for the ^2^L_s_ states of **5^+^**–**7^+^** (see Supporting Information) [27].

It can be seen that for states where the electron and hole size increase faster than the exciton size, the correlation coefficient is larger and increases faster. In the case of ^1^L_w_, ^2^L_w_ and ^2^I_b_, where the electron and hole size and exciton size increase equally with growing acene length, the correlation coefficient is small and increases only slightly. For states like ^2^L_s_ and ^2^B_b_, where the electron and hole size increase faster than the exciton size, the correlation coefficient is large and increases faster. The exciton size can be computed for localized excited states based on the hole and electron size and correlation coefficient as $d_{exc}=\sqrt{\sigma_{h}^{2}+\sigma_{e}^{2}-2R_{eh}\sigma_{h}\sigma_{e}}$, which explains the observed relation of these exciton properties [22].

**FIGURE 5:**
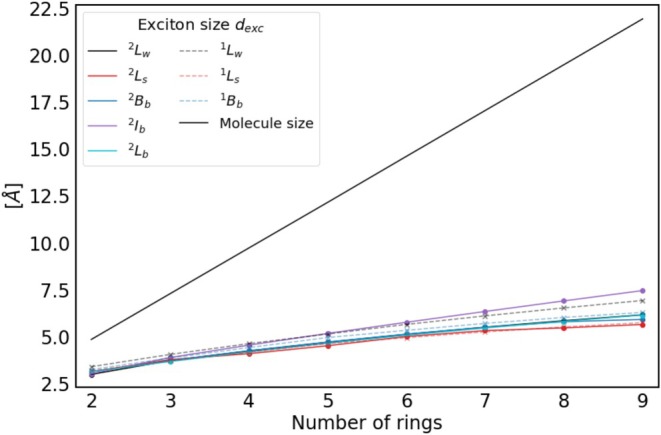
Exciton size ($d_{exc}$) of all investigated excited states of **2**–**9** and **2^+^**–**9^+^** compared to the molecular size along the long axis calculated using CAM‐B3LYP/6‐311G*.

Finally, set into relation to the spatial size of the molecules in Figure 5, it can be seen that the exciton size grows much more slowly than the molecular size along the long molecular axis. The molecular size corresponds essentially to the size of the involved π and $\pi^{*}$ orbitals, which are fully delocalized and thus extend over the complete molecules. In Figure 5, the difference between the size of the molecular orbitals describing the transition and the exciton size becomes apparent. In fact, molecular orbitals describe the uncorrelated probability of finding the hole and the electron at a certain location in the molecule. Since they are completely delocalized over the whole molecule, this probability is the same everywhere. The exciton size, on the other hand, is the expectation value for the actual spatial extent of the exciton and is therefore smaller. It can also be noted that the absolute size and the trend of the change in exciton size seem to be very similar between the different excited states, especially in relation to the change in molecular size.

### 4.2 Molecular Plasmons in Acenes and Acene Cations

By employing the procedure introduced in Section 2.2, it was shown that linear polyenes possess excited states, which can be characterized as molecular plasmons. It was further pointed out that this procedure should also be applicable to non‐linear molecular systems, like acenes, if a suitable electron gas model is used [24]. As discussed in Section 2.2, molecular plasmons are collective excitations described by linear combinations of *m* possible single‐particle transitions with a given momentum transfer **q** that gather all the absorption intensity of the other excited states described by the same transitions. (For one set of *m* transitions with a certain **q**, there is one plasmonic excitation and *m*‐1 single‐particle excitations.) These criteria are possibly fulfilled by the ^1^B_b_ state of neutral acenes. It is a linear combination of two particle‐hole transitions and collects all the intensity of the lower‐energy ^1^L_s_ state, which is described by the same transitions (see Table 1). To fulfill these criteria, the two transitions need to have the same momentum transfer.

**TABLE 1:** Excitation energies [eV], oscillator strength and main orbital contributions of ^1^L_s_ and ^1^B_b_ of **3**, calculated using CAM‐B3LYP/6‐311G*.

State	E [eV]	f_ocs_	Main contrib. (amplitude)
^1^L_s_	4.19	0.00	H−1→L (0.70)
			H→L+1 (0.69)
^1^B_b_	5.97	3.19	H−1→L (−0.68)
			H→L+1 (0.69)

Considering the π‐electrons as a one‐dimensional electron gas in a box, the momentum transfer of a particular single‐particle transition is related to the corresponding change in the number of nodes of the participating orbitals. However, in the case of acenes, a two‐dimensional system has to be considered. The wavevectors assigned to the frontier molecular orbitals will therefore have two components, in x‐ and y‐directions, and the momentum transfer of a particular particle‐hole transition will be classified with respect to the change in number of nodes in both directions. Here, the x‐ and y‐axes are placed along the long and short molecular axes, respectively, to conform with the orientation adopted in the calculations.

**FIGURE 6:**
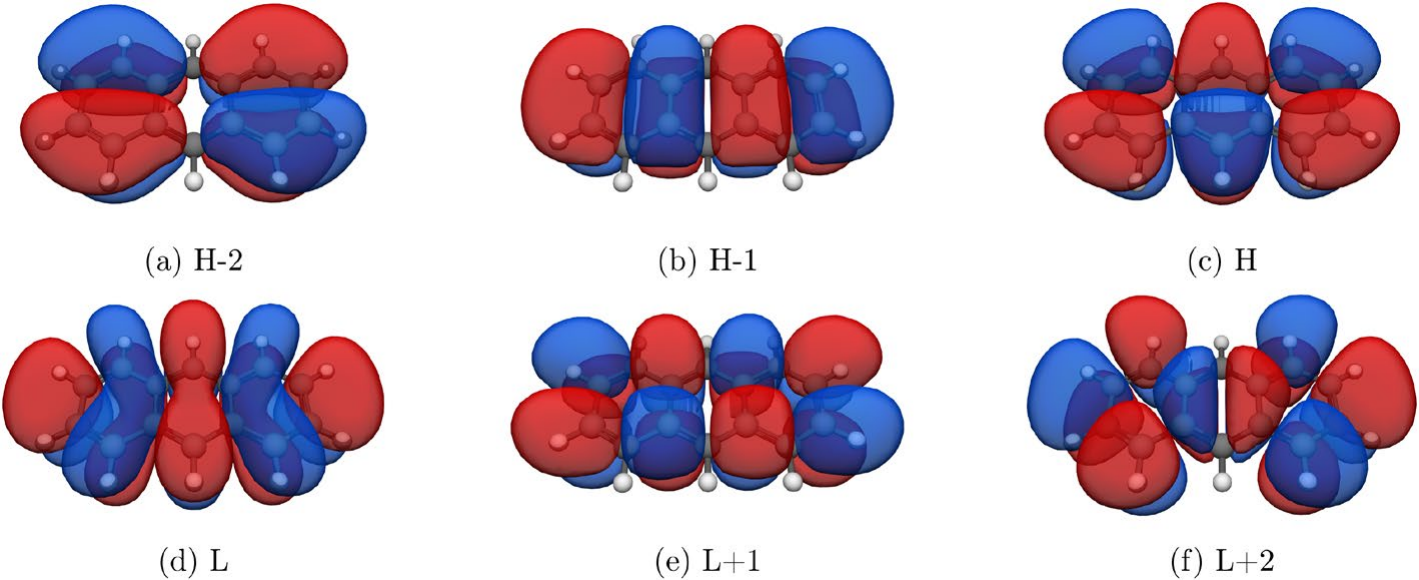
Frontier molecular orbitals H−2, H−1, H, L, L+1 and L+2 of **3** obtained at CAM‐B3LYP/6‐311G* and visualized using IQmol. (a) H−2, (b) H−1, (c) H, (d) L, (e) L+1, and (f) L+2.

The changes in the number of nodes of the relevant transitions for the ^1^B_b_ state of **3** and **5** are analyzed in the following. The frontier molecular orbitals of **3** from H‐2 to L+2 are shown in Figure 6.

The total number of nodes of these orbitals N (including those in z‐direction), the number of nodes in x‐ and y‐directions $N_{x}$ and $N_{y}$ and the change in number of nodes for all particle‐hole transitions involving these orbitals (ΔN, $\Delta N_{x}$ and $\Delta N_{y}$) are shown in Table 2.

**TABLE 2:** Number of nodes N in selected molecular orbitals of **3** along the x‐ and y‐axes and change in number of nodes for specific transitions.

MO	N	$N_{x}$	$N_{y}$
L+2	6	5	0
L+1	5	3	1
L	5	4	0
H	4	2	1
H−1	4	3	0
H−2	3	1	1

Transition	ΔN	$\Delta N_{x}$	$\Delta N_{y}$
H→L	1	2	1
H−1→L	1	1	0
H→L+1	1	1	0
H−2→L	2	3	−1
H→L+2	2	3	−1

Looking at the overall change in the number of nodes per orbital, the transitions H‐L, H‐L+1, and H‐1‐L all have a change in nodes of 1. When evaluating the change in number of nodes in x‐ and y‐directions separately, the transition H‐L has $\Delta N_{x}$ = 2 and $\Delta N_{y}$ = 1, while H‐L+1 and H‐1‐L both have $\Delta N_{x}$ = 1 and $\Delta N_{y}$ = 0. Therefore, under the approximation of acenes as two‐dimensional systems, the ^1^B_b_ state *is* a linear combination of all replacements of a certain momentum transfer. It should be noted that the number of nodes along the z‐axis does not change for the relevant orbitals, which supports the assumption that a two‐dimensional analysis is justified. In the following, the corresponding analysis is done for **5**.

The transitions that contribute to the ^1^L_s_ and ^1^B_b_ of **5** are H‐L+2 and H‐2‐L, which can be seen in Table 3. It was previously observed that this change occurs, using the current method, when going from **4** to **5** and again when going from **7** to **8** to H‐L+3 and H‐3‐L [27].

**TABLE 3:** Excitation energies [eV], oscillator strength and main orbital contributions of ^1^L_s_ and ^1^B_b_ of **5**, calculated using CAM‐B3LYP/6‐311G*.

State	E [eV]	f_ocs_	Main contrib. (amplitude)
^1^L_s_	3.47	0.00	H−2→L (0.71)
			H→L+2 (−0.67)
^1^B_b_	4.89	5.44	H−2→L (0.67)
			H→L+2 (0.71)

Table 4 shows that the change in nodes for the transition H+2‐L and H‐2‐L are $\Delta N_{x}$ = 1 and $\Delta N_{y}$ = 0, the same as for H‐L+1 and H‐1‐L in **3** and that there are no other transition with the same momentum transfer. The frontier molecular orbitals can be seen in Figure S9 in the Supporting Information. The ^1^B_b_ of **5** therefore also fulfills the criteria for a molecular plasmon. This can be analogously shown for the ^1^B_b_ state of all investigated acenes.

**TABLE 4:** Number of nodes N in the selected molecular orbitals of **5** along the x‐ and y‐axes and change in number of nodes for specific transitions.

MO	N	$N_{x}$	$N_{y}$
L+2	7	5	1
L+1	8	7	0
L	7	6	0
H	6	4	1
H−1	5	3	1
H−2	6	5	0

Transition	ΔN	$\Delta N_{x}$	$\Delta N_{y}$
H→L	1	2	−1
H−1→L	2	3	−1
H→L+1	2	3	−1
H−2→L	1	1	0
H→L+2	1	1	0

**FIGURE 7:**
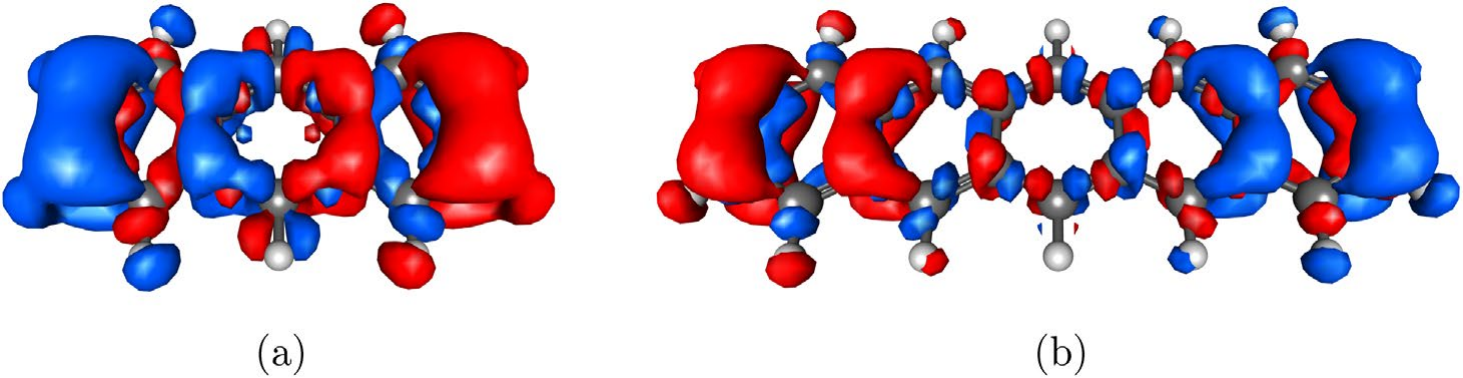
Transition densities of the ^1^B_b_ state of **3** (a) and **5** (b) obtained using CAM‐B3LYP/6‐311G* and visulaized using IQmol.

Another characteristic feature of molecular plasmons is the nodal structure of their transition densities. This can be observed for the ^1^B_b_ state of **3** and **5** in Figure 7. The transition density nicely displays the collective oscillation of the electron density from one end of the acene to the other along the long molecular axis.

This analysis is now extended to the excited states of acene cations for the example of **3^+^** and for the first time to an open‐shell molecule. Here, the ^2^B_b_ state is the obvious candidate for a plasmonic excitation (see Table 5).

**TABLE 5:** Excitation energies [eV], oscillator strength and main orbital contributions of ^2^I_b_, ^2^L_b_, ^2^L_s_, and ^2^B_b_ of **3^+^**, calculated using CAM‐B3LYP/6‐311G*. Only contributions with $\Delta N_{x}$ = 1 and $\Delta N_{y}$ = 0 are included here. Omitted contributions have amplitudes below 0.15.

State	E [eV]	f_ocs_	Relevant contrib. (amplitude)
^2^I_b_	2.19	0.15	αH→αL+1 (−0.13)
			αH−1→αL (−0.24)
			βH→βL (0.95)
			βH−1→βL+1 (0.06)
^2^L_b_	3.67	0.12	αH→αL+1 (−0.2)
			αH−1→αL (0.8)
			βH→βL (−0.15)
			βH−1→βL+1 (0.44)
^2^L_s_	4.44	0.00	αH→αL+1 (0.74)
			αH−1→αL (−0.16)
			βH→βL (0.02)
			βH−1→βL+1 (0.62)
^2^B_b_	5.80	2.74	αH→αL+1 (−0.59)
			αH−1→αL (−0.41)
			βH→βL (−0.21)
			βH−1→βL+1 (0.61)

Since we are looking at an open‐shell system, α‐ and β‐orbitals are no longer equivalent, and the number of nodes and change of number of nodes have to be evaluated for α‐ and β‐orbitals separately. The frontier molecular orbitals can be seen in Figure S10 in the Supporting Information. Table 6 shows the total number of nodes N, the number of nodes along x‐ and y‐directions $N_{x}$ and $N_{y}$ and the respective change in number of nodes of all possible transitions between the orbitals in total and along the x‐ and y‐axes (ΔN, $\Delta N_{x}$, $\Delta N_{y}$).

**TABLE 6:** Number of nodes N in the selected molecular orbitals of **3^+^** along the x‐ and y‐axes and change in number of nodes for specific transitions.

	α			β		
MO	N	$N_{x}$	$N_{y}$	N	$N_{x}$	$N_{y}$
L+2	6	5	0	5	3	1
L+1	5	3	1	5	4	0
L	5	4	0	4	2	1
H	4	2	1	3	1	1
H−1	4	3	0	4	3	0
H−2	3	1	1	2	0	1

transition	ΔN	$\Delta N_{x}$	$\Delta N_{y}$	ΔN	$\Delta N_{x}$	$\Delta N_{y}$
H→L	1	2	−1	1	1	0
H−1→L	1	1	0	0	−1	1
H→L+1	1	1	0	2	3	−1
H−2→L	2	3	−1	2	2	0
H→L+2	2	3	−1	2	2	0
H−1→L+1	1	0	1	1	1	0
H−1→L+2	2	2	0	1	0	1
H−2→L+1	2	2	0	3	4	−1
H−2→L+2	3	4	−1	3	3	0

**FIGURE 8:**
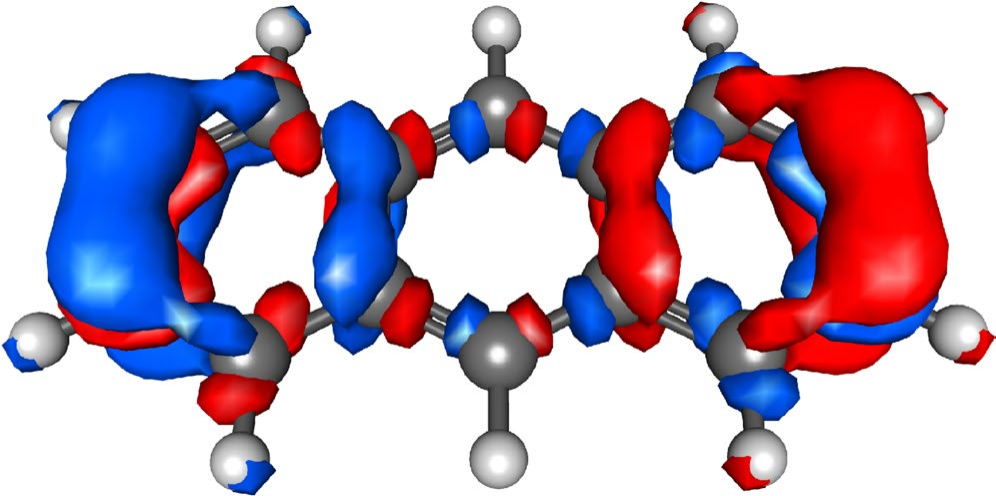
Transition densities of the ^2^B_b_ state of **3^+^**. Calculated by averaging the α‐ and β‐transition densities ($\frac{1}{2}(\rho_{\alpha }+\rho_{\beta })$) obtained using CAM‐B3LYP/6‐311G* and visulaized using IQmol.

In the open‐shell molecule **3^+^** there are now four transitions, which show a change of nodes along the x‐ and y‐axes of $\Delta N_{x}$ = 1 and $\Delta N_{y}$ = 0: αH→αL+1, *α*H‐1→*α*L, βH→βL and βH−1→βL+1. It was stated previously that for *m* contributing single‐particle transitions with a given momentum transfer **q**, there is one plasmonic excitation and *m*‐1 non‐plasmonic excited states made up of the same transitions [24]. Since in the case of **3^+^**, there are four transitions with the same momentum transfer, there should also be four excited states made up of these transitions. Table 5 shows that these are the excited states ^2^I_b_, ^2^L_b_, ^2^L_s_, and ^2^B_b_; however, this only becomes apparent when also very small contributions are considered. The ^2^B_b_ state of **3^+^** therefore also fulfills the criteria for a plasmonic state. It can also be concluded that the ^2^I_b_, ^2^L_b_, ^2^L_s_, and ^2^B_b_ excited states are in the same relation to each other as the ^1^L_s_ and ^1^B_b_ states of **3**. The α‐ and β‐transition densities of ^2^B_b_ of **3^+^** were averaged to give the total transition density in Figure 8, which is very similar to the one of ^1^B_b_ of **3**. The collective oscillation of the electron density along the long molecular axis is clearly reflected in the transition density, which shows that molecular plasmons are also present in open‐shell radical cations. However, our analysis shows that there are no new lower‐lying bright plasmonic excitations in the acene cations compared to the neutrals, since these are the same states in both, which is in contrast to other studies stating that acene cations show an additional lower‐energy plasmon [33, 45, 46]. Nonetheless, these studies show that there are applications for acene cations in devices due to their red‐shifted absorption spectrum [33, 45, 46].

## 5 Summary

Exciton properties of the excited states ^1^L_w_, ^1^L_s_, ^1^B_b_ of neutral acenes and ^2^I_b_, ^2^L_w_, ^2^L_s_, ^2^L_b_ and ^2^B_b_ of acene cations are investigated for **2**–**9** and **2^+^**–**9^+^**. For both neutral and cationic acenes, the exciton, hole, and electron size increase with increasing acene length, where the exciton size is generally larger for the excited states of the neutral acenes. While for the neutral acenes the electron size is always larger than the hole size and both increase equally with increasing acene length, the hole size grows faster than the electron size for the cations, so that it is larger for all the longer acenes. It is observed that for excited states ^1^L_w_, ^2^L_w_ and ^2^I_b_, the hole and electron size increase similarly to the exciton size, while for the other states, the hole and electron size increase faster than the exciton size with growing acene length. This can be linked to the development of the correlation coefficient, which increases slowly for the former states and faster for the latter ones. The exciton size of all excited states is set into relation to the size of the molecular orbitals, which shows that the exciton size increases much more slowly and converges towards a certain value. While the exciton size is the spatial extension of the exciton computed as the expectation value of the exciton wavefunction, the molecular orbitals simply display the uncorrelated probability of finding the hole and particle somewhere in the molecule.

The ^1^B_b_ excited states of **3** and **5** and the ^2^B_b_ excited state of **3** ^+^ are analyzed with respect to specific plasmon characteristics. This is done by analyzing the momentum transfer of participating single‐particle transitions based on the change in the number of the corresponding orbitals, as previously done for polyenes. By applying this procedure to acenes as two‐dimensional systems, it can be shown that these states correspond to molecular plasmons with a characteristic and very similar “collective” transition density for **3**, **5** and **3^+^**. Since the plasmon of **3^+^** is essentially inherited from the neutral **3**, this contrasts previous claims that acene cations possess additional low‐lying plasmons [33, 45, 46].

## Conflicts of Interest

The authors declare no conflicts of interest.

## Supporting information




**Data S1**: Supporting Information.

## Data Availability

The data that support the findings of this study are available from the corresponding author upon reasonable request.
