# Supplementary material for: Molecular Excitons and Plasmons in Acenes and Their Radical Cations
Source: J Comput Chem. 2025 Aug 18;46(23):e70211. doi: 10.1002/jcc.70211 (PMC12360141; doi:10.1002/jcc.70211)
Supplement: Supplementary file 1 — Data S1: Supporting Information. [file JCC-46-0-s001.pdf]

Supporting Information - Molecular Excitons and Plasmons in Acenes and Acene Cations

Contents

1 **Electronic Excitations of Acenes and Acene Radical Cations 2-12 and 2^+-12^+** 1

2 **Exciton Properties of Acenes and Acene Cations** 1

3 **Exciton Properties of 2-12 and 2^+-12^+** 4

4 **Frontier Molecular Orbitals of 5 and 3^+** 6

1 Electronic Excitations of Acenes and Acene Radical Cations 2-12 and 2^+-12^+

Table S1: Excitation energies [eV] of low-lying excited states of **2-12** and **2^+-12^+** calculated using the CAM-B3LYP functional.

	2	3	4	5	6	7	8	9	10	11	12
^1L_w	5.02	3.92	3.17	2.41	2.25	1.96	1.74	1.57	0.48	0.53	0.56
^1L_s	4.77	4.19	3.82	3.47	3.38	3.24	3.14	3.06	1.10	1.04	0.98
^1B_b	6.78	5.97	5.39	4.89	4.62	4.35	4.14	3.96	2.72	2.58	2.45
	2+	3+	4+	5+	6+	7+	8+	9+	10+	11+	12+
^2I_b	2.36	2.19	1.96	1.76	1.60	1.46	1.34	1.24	1.29	1.21	1.13
^2L_w	4.00	2.71	2.11	1.65	1.31	1.04	0.84	0.67	0.32	0.23	0.15
^2L_b	4.18	3.67	3.32	3.08	2.90	2.77	2.66	2.58	2.43	2.39	2.36
^2L_s	4.99	4.44	4.12	3.86	3.67	3.58	3.47	3.39	3.31	3.25	3.19
^2B_b	6.58	5.80	5.25	4.83	4.51	4.25	4.05	3.87	3.74	3.62	3.52

2 Exciton Properties of Acenes and Acene Cations

In **Figure S1** the exciton, hole and electron size of ^1L_s and ^2L_s for **2-9** and **2^+-9^+** are shown. For the neutral acenes, the exciton size increases from 3.2 Å for **2** to 5.7 Å for **9**. The hole and electron size increase as well starting from 2.3 Å and 2.4 Å for **2** respectively and both going to 5.2 Å for **9**. The exciton size of the ^2L_s state increases from 3.1 Å for **2^+** to 5.6 Å for **9^+**. The hole and electron size are similar for **2^+** with 2.3 Å and 2.4 Å respectively and the hole size increases stronger than the electron size with 6.5 Å and 5.8 Å respectively for **9^+**, as observed for ^2L_w. The trend displayed by the exciton properties of ^2L_s is unsteady for the molecules **5^+** to **7^+**. An explanation for this could be that the ^2L_s state mixes with a state described by the transition βH-4$\rightarrow\beta$L for **4^+-7^+**,

which influences its exciton properties.
The exciton properties of the ^1B_b and ^2B_b states of **2-9** and **2**$^+$-**9**$^+$ are shown in **Figure S2**. The trend looks similar to that of the ^1L_s and ^2L_s states. For the neutral acenes, the exciton size of ^1B_b increases with growing acene length from 3.2 Å for **2** to 6.3 Å for **9**. The hole and electron size are similar and are increasing for **2-9** from 2.3 Å and 2.4 Å respectively to 5.7 Å and 5.8 Å respectively. For the ^2B_b state, the exciton size increases from 3.1 Å for **2**$^+$ to 5.9 Å for **9**$^+$. As previously observed, the hole size increases more strongly than the electron size from 2.3 Å and 2.4 Å respectively for **2**$^+$ to 7.0 Å and 6.1 Å respectively for **9**$^+$.
Figure S3 shows the exciton properties of the excited state ^2L_b of **2**$^+$-**9**$^+$. The exciton size increases from 3.1 Å for **2**$^+$ to 6.1 Å for **9**$^+$. As observed for the other cation excited states, the hole size grows more strongly than the electron size, starting from 2.3 Å and 2.4 Å for hole and electron size for **2**$^+$ and ending at 6.0 Å and 5.1 Å for **9**$^+$.

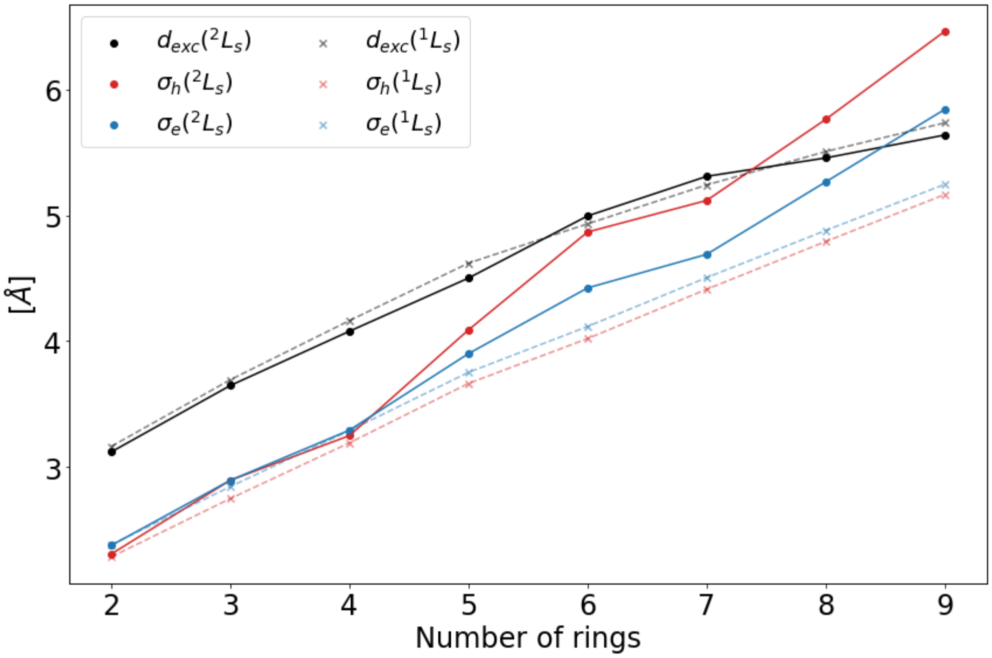

Figure S1: Exciton (d_{exc}), hole (σ_h) and electron size (σ_e) of the excited states ^1L_s of **2-9** and ^2L_s of **2**$^+$-**9**$^+$ calculated using CAM-B3LYP/6-311G*.

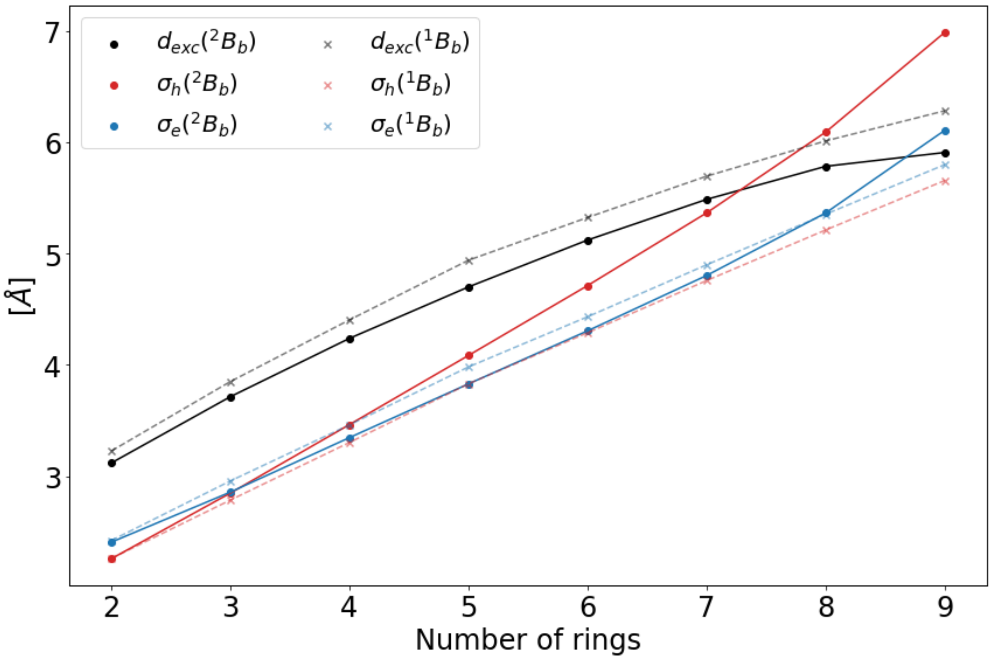

Figure S2: Exciton (d_{exc}), hole (σ_h) and electron size (σ_e) of the excited states $^1\mathrm{B_b}$ of **2**-**9** and $^2\mathrm{B_b}$ of $\mathbf{2}^+$-$\mathbf{9}^+$ calculated using CAM-B3LYP/6-311G*.

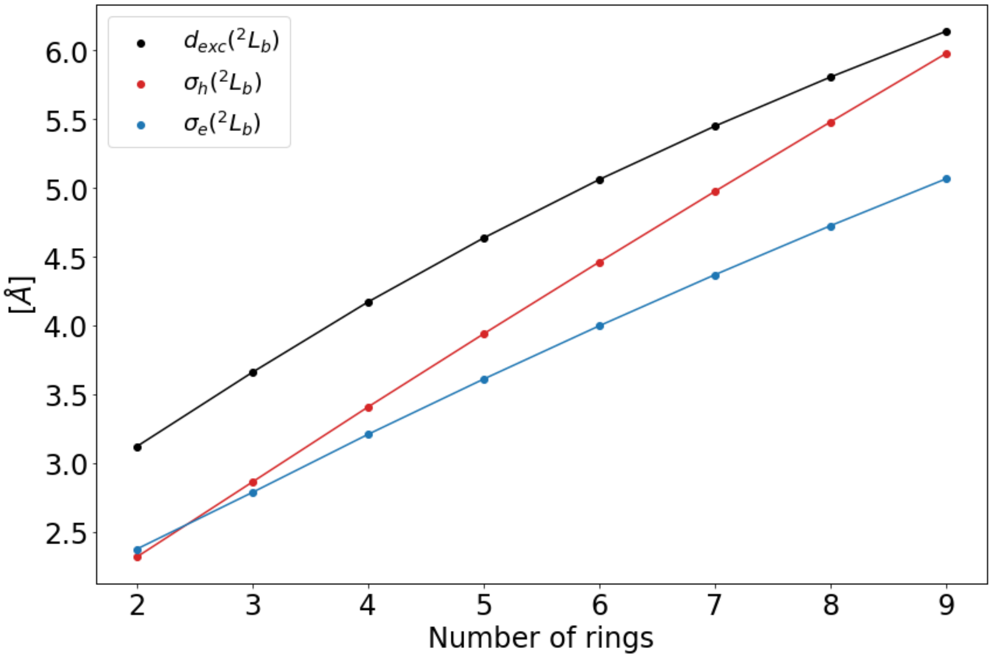

Figure S3: Exciton (d_{exc}), hole (σ_h) and electron size (σ_e) of the excited states $^2\mathrm{L_b}$ of $\mathbf{2}^+$-$\mathbf{9}^+$ calculated using CAM-B3LYP/6-311G*.

3 Exciton Properties of 2-12 and 2^+-12^+

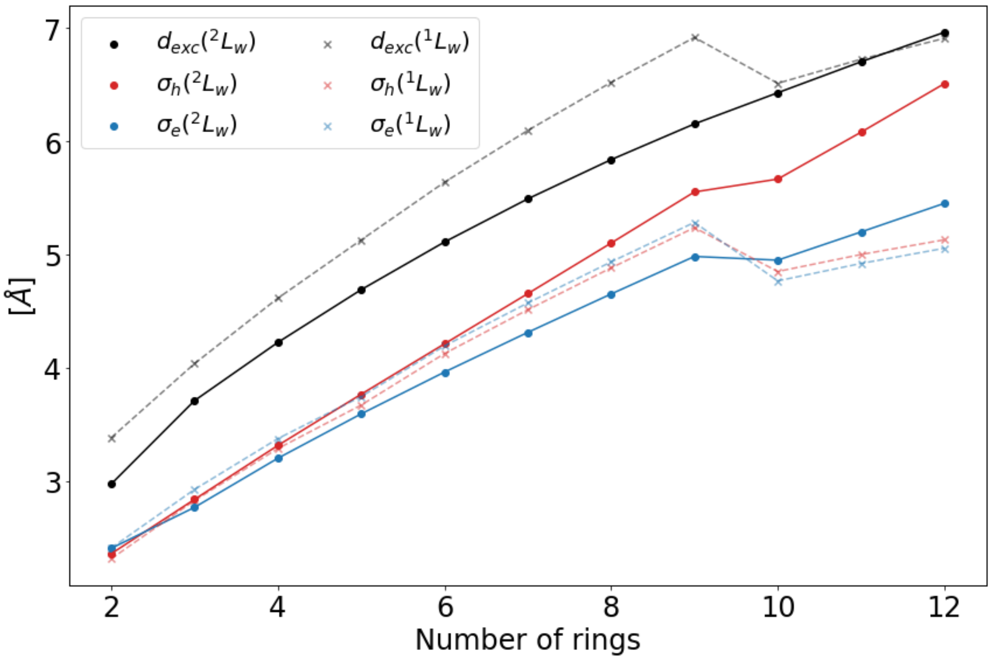

Figure S4: Exciton (d_{exc}), hole (σ_h) and electron size (σ_e) of the excited states ^1L_w of **2-12** and ^2L_w of **2^+-12^+** calculated using CAM-B3LYP/6-311G*.

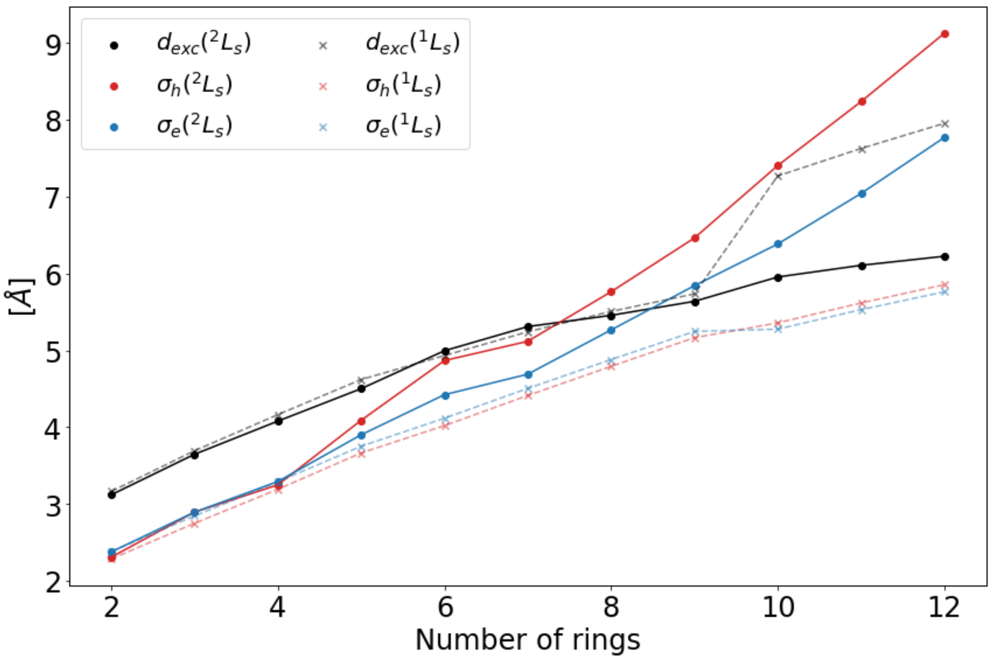

Figure S5: Exciton (d_{exc}), hole (σ_h) and electron size (σ_e) of the excited states ^1L_s of **2-12** and ^2L_s of **2^+-12^+** calculated using CAM-B3LYP/6-311G*.

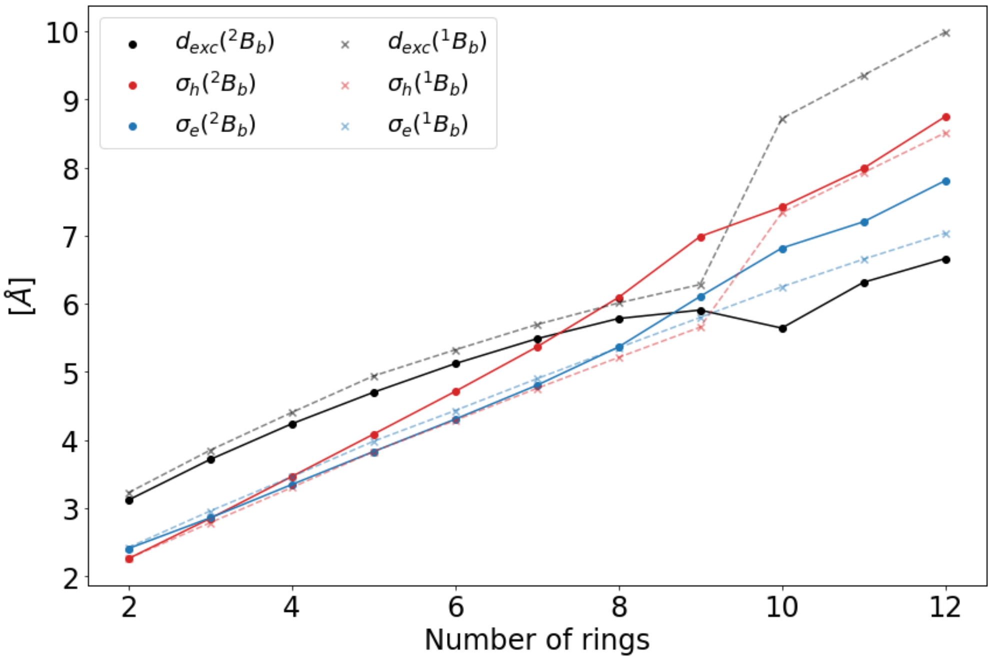

Figure S6: Exciton (d_{exc}), hole (σ_h) and electron size (σ_e) of the excited states $^1\mathrm{B_b}$ of **2-12** and $^2\mathrm{B_b}$ of $\mathbf{2}^+$-$\mathbf{12}^+$ calculated using CAM-B3LYP/6-311G*.

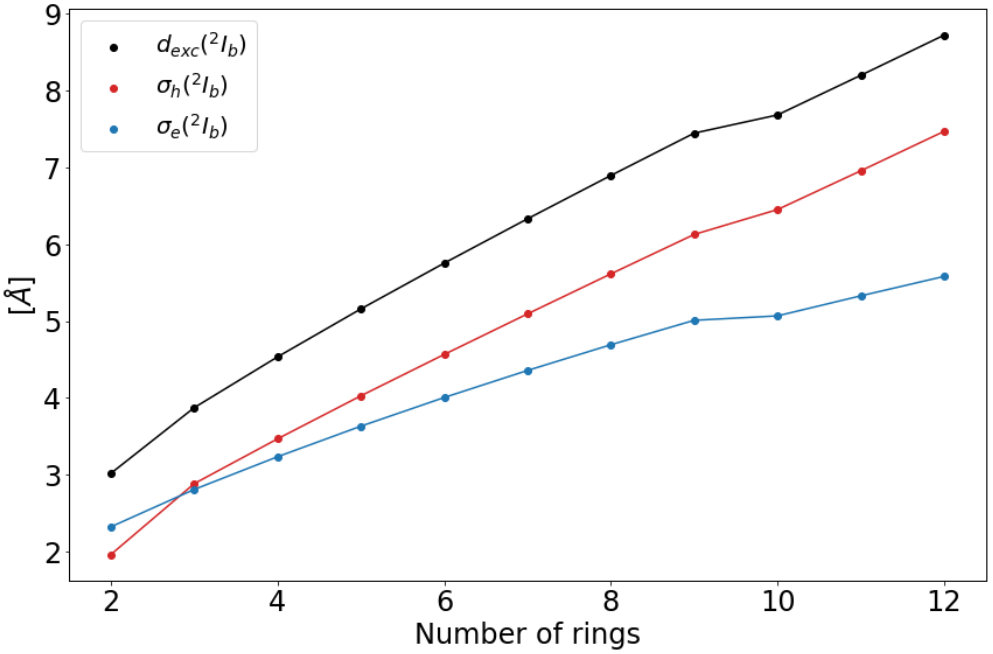

Figure S7: Exciton (d_{exc}), hole (σ_h) and electron size (σ_e) of the excited states $^2\mathrm{I_b}$ of $\mathbf{2}^+$-$\mathbf{12}^+$ calculated using CAM-B3LYP/6-311G*.

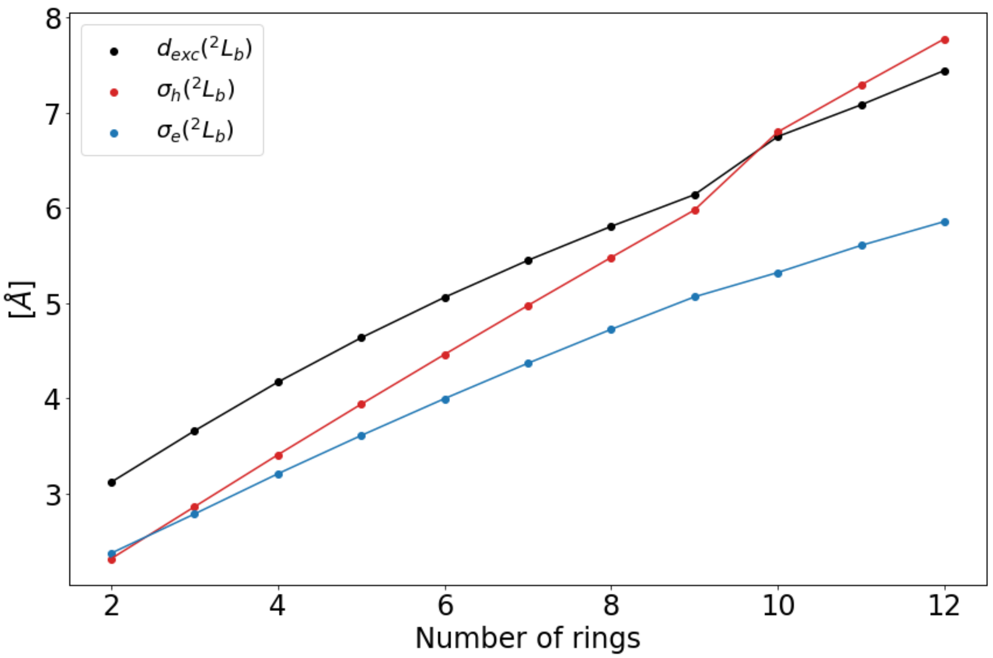

Figure S8: Exciton (d_{exc}), hole (σ_h) and electron size (σ_e) of the excited states $^2\mathrm{L_b}$ of **2**$^+$-**12**$^+$ calculated using CAM-B3LYP/6-311G*.

4 Frontier Molecular Orbitals of 5 and 3$^+$

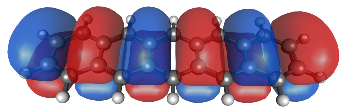

(a) H-2

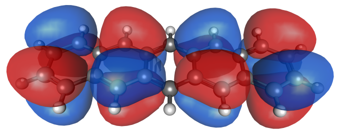

(b) H-1

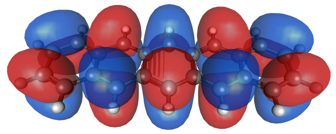

(c) H

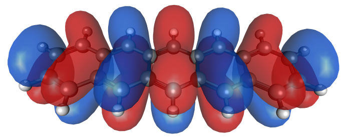

(d) L

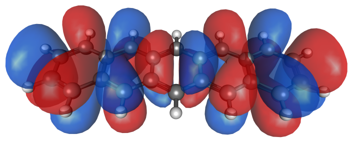

(e) L+1

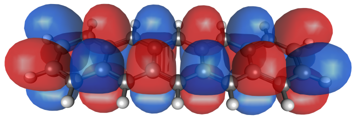

(f) L+2

Figure S9: Frontier molecular orbitals H-2, H-1, H, L, L+1 and L+2 of **5** obtained at CAM-B3LYP/6-311G* and visualized using IQmol.

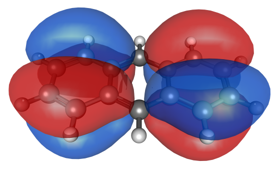

(a) α-H-2

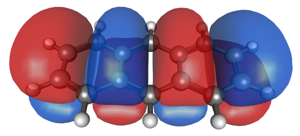

(b) α-H-1

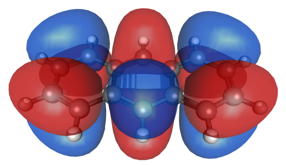

(c) α-H

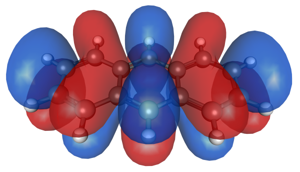

(d) α-L

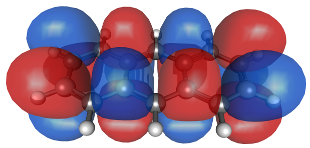

(e) α-L+1

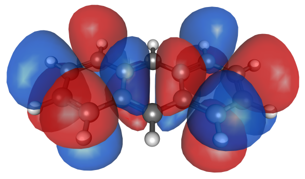

(f) α-L+2

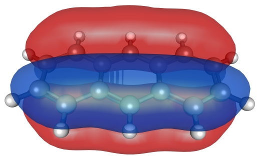

(g) β-H-2

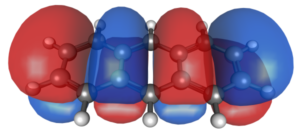

(h) β-H-1

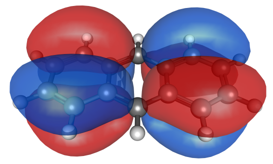

(i) β-H

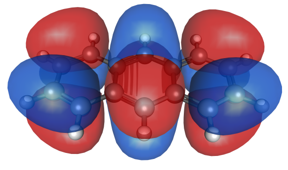

(j) β-L

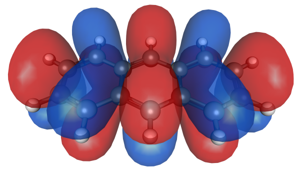

(k) β-L+1

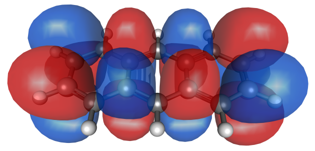

(l) β-L+2

Figure S10: Frontier molecular orbitals α- and β-H-2, H-1, H, L, L+1 and L+2 of $\mathbf{3}^+$ obtained at CAM-B3LYP/6-311G* and visualized using IQmol.
